# Effectiveness of repellent delivered through village health volunteers on malaria incidence in villages in South-East Myanmar: a stepped-wedge cluster-randomised controlled trial protocol

**DOI:** 10.1186/s12879-018-3566-y

**Published:** 2018-12-14

**Authors:** Win Han Oo, Julia C. Cutts, Paul A. Agius, Kyaw Zayar Aung, Poe Poe Aung, Aung Thi, Nyi Nyi Zaw, Htin Kyaw Thu, Wai Yan Min Htay, Ricardo Ataide, Katherine O’Flaherty, Ai Pao Yawn, Aung Paing Soe, James G. Beeson, Brendan Crabb, Naanki Pasricha, Freya J. I. Fowkes

**Affiliations:** 1 Burnet Institute, Melbourne, Australia; 2 Burnet Institute, Yangon, Myanmar; 3 Department of Epidemiology and Preventive Medicine, Monash University, Melbourne, Australia; 4 Judith Lumley Centre, La Trobe University, Melbourne, Australia; 5 Department of Public Health, Myanmar Ministry of Health and Sport, Nay Pyi Taw, Myanmar; 6 Centre for Epidemiology and Biostatistics, Melbourne School of Population and Global Health, University of Melbourne, Melbourne, Australia; 7 Department of Medicine, University of Melbourne, Melbourne, Australia; 8 Department of Microbiology and Central Clinical School, Monash University, Melbourne, Australia

**Keywords:** Malaria, Repellent, Mosquito, Plasmodium

## Abstract

**Background:**

To combat emerging drug resistance in the Greater Mekong Sub-region (GMS) the World Health Organization and GMS countries have committed to eliminating malaria in the region by 2030. The overall approach includes providing universal access to diagnosis and treatment of malaria, and sustainable preventive measures, including vector control. Topical repellents are an intervention that can be used to target residual malaria transmission not covered by long lasting insecticide nets and indoor residual spraying. Although there is strong evidence that topical repellents protect against mosquito bites, evidence is not well established for the effectiveness of repellents distributed as part of malaria control activities in protecting against episodes of malaria. A common approach to deliver malaria services is to assign Village Health Volunteers (VHVs) to villages, particularly where limited or no services exist. The proposed trial aims to provide evidence for the effectiveness of repellent distributed through VHVs in reducing malaria.

**Methods:**

The study is an open stepped-wedge cluster-randomised controlled trial randomised at the village level. Using this approach, repellent (N,N-diethyl-benzamide – 12% *w*/w, cream) is distributed by VHVs in villages sequentially throughout the malaria transmission season. Villages will be grouped into blocks, with blocks transitioned monthly from control (no repellent) to intervention states (to receive repellent) across 14 monthly intervals in random order). This follows a 4-week baseline period where all villages do not receive repellent. The primary endpoint is defined as the number of individuals positive for *Plasmodium falciparum* and *Plasmodium vivax* infections diagnosed by a rapid diagnostic test. Secondary endpoints include symptomatic malaria, Polymerase Chain Reaction (PCR)-detectable *Plasmodium* spp. infections, molecular markers of drug resistance and antibodies specific for *Plasmodium* spp. parasites.

**Discussion:**

This study has been approved by relevant institutional ethics committees in Myanmar and Australia. Results will be disseminated through workshops, conferences and peer-reviewed publications. Findings will contribute to a better understanding of the optimal distribution mechanisms of repellent, context specific effectiveness and inform policy makers and implementers of malaria elimination programs in the GMS.

**Trial registration:**

Australian and New Zealand Clinical Trials Registry (ACTRN12616001434482). Retrospectively registered 14th October 2016.

**Electronic supplementary material:**

The online version of this article (10.1186/s12879-018-3566-y) contains supplementary material, which is available to authorized users.

## Background

Malaria, a disease caused by *Plasmodium* spp. and transmitted by *Anopheline* mosquitoes, remains a significant cause of morbidity and mortality worldwide [1]. Tools to reduce the burden of malaria have largely focused on effective antimalarials, currently Artemisinin-based Combination Therapies (ACT), and prevention of infection through long-lasting insecticidal nets (LLINs), and indoor residual spraying (IRS) with insecticides. Upscaling of both of these highly efficacious interventions in the past decade has led to a significant reduction in malaria morbidity and mortality globally [1, 2].

Artemisinin resistant falciparum malaria, defined by the presence of mutations in the *kelch13* gene and a slow clearance phenotype, is now firmly established in the Greater Mekong Subregion (GMS) [3]. To combat the spread of resistance the World Health Organization (WHO) and GMS countries have committed to eliminating malaria in the region by 2030. The overall approach includes providing universal access to diagnosis and treatment of malaria, and sustainable preventive measures, including vector control [4]. This includes universal coverage of all at-risk populations with LLINs or IRS and supplementary measures where appropriate (e.g. larval source management, repellents) [4]. Supplementary measures are particularly pertinent in the GMS, because while LLINs and IRS target mosquitoes resting or biting indoors at night, many malaria vectors in the region readily feed outdoors [5], potentially rendering malaria control interventions like LLINs and indoor residual spraying less effective. Moreover, there is a particular need for personal protection targeting groups most at risk of malaria in the GMS, namely mobile workers and migrant populations and those residing in or near forested areas [4].

Topical repellents are an intervention that can be used to target residual malaria transmission not covered by LLIN and IRS. Local malaria vectors in Kayin, Kayah and Bago (East) include *An. dirus* complex and *An. minimus* complex, which are classified as “late biters”. Although there is strong evidence that topical repellents protect against mosquito bites [6], evidence for the effectiveness of repellents in protecting against episodes of malaria is not well established. A recent systematic review identified 10 trials assessing the efficacy of topical insect repellents against malaria [7], and meta-analysis indicated that topical repellents showed an 18% protective efficacy against *P. falciparum* malaria, but with wide confidence intervals (95% CI: -8%, 38%) [7]. Only two of these trials were conducted in South-East Asia (Laos and Thailand) [8, 9] with a further trial recently reported in Cambodia in 2016 [10]. None of these South-East Asian studies demonstrated effectiveness of repellent against malaria incidence. There is considerable heterogeneity between trials including study design, active ingredients, concentration and formulation of the repellent, user compliance, co-interventions, outcome measures, participants, and repellent distribution methods and further well-designed trials of topical repellents are required to establish their real-world effectiveness [7].

In Myanmar, mosquito repellents have been introduced by the private sector for the prevention of mosquito-borne diseases. However, the Ministry of Health is yet to approve the distribution of topical repellents as part of large-scale disease prevention programs in Myanmar as there is not enough evidence for their effectiveness. In particular, information is needed on how best to distribute repellents in hard to reach and isolated groups (e.g. those in conflict/ceasefire areas). In Myanmar, and other GMS countries, a common approach is to assign Village Health Volunteers (VHVs) to villages where limited or no services exist, with the aim of providing malaria prevention, diagnosis, treatment and referral services where necessary. Evidence is needed for the effectiveness of personal insect repellent distributed to villages through VHV as a component of artemisinin resistance containment and malaria control programs in the GMS.

The primary objective of this trial is to determine the effectiveness of distributing repellent to villages through VHV in high risk geographically isolated populations to reduce the incidence of *P. falciparum* and *P. vivax* infections. Secondary objectives are to understand the broader implications of personalised malaria control measures, in particular on the presence of molecular-detected *P. falciparum* and *P. vivax* parasites, parasite mutations that confer drug resistance (due to decreased drug pressure in the effective intervention arm) and its impact on naturally-acquired immunity to malaria. This study is novel in that it will determine the effectiveness of repellents distributed to villages through VHVs in reducing malaria in geographically isolated, high risk populations. It will also be the first to examine the effectiveness of repellents on the frequency of parasite drug resistance mutations and immunity as additional markers of malaria exposure. The proposed study will provide an evidence base for the use of repellents in malaria control and elimination programs in the GMS.

## Methods/design

This study protocol adheres to the SPIRIT (Standard Protocol Items: Recommendations for Interventional Trials) statement (Additional file 1). This protocol is version 15 dated 2 March 2015.

### Trial registration

This trial was retrospectively registered with the Australian and New Zealand Clinical Trials Registry (ACTRN12616001434482) on 14 October 2016.

### Research objectives and endpoints

The overall aim of the trial is to assess the effectiveness of personal insect repellent distributed to villages through VHV in malaria control and elimination programs for artemisinin resistance containment in Myanmar.

### Primary research objective and hypothesis

The primary research objective is to evaluate the effectiveness of personal insect repellent (N,N-diethyl-benzamide – 12% w/w, cream) [11] distributed by VHV to high risk village groups including mobile, migrant, and forest workers living in South-East Myanmar in preventing *P. falciparum and P. vivax* infections. The primary study hypothesis is that incidence of *Plasmodium* spp. infection will, on average, be lower in study villages once personal insect repellent is distributed by VHV. The primary endpoint is incidence of *P. falciparum* and *P. vivax* infections; defined as the number of individuals in the village positive for *P. falciparum and P. vivax* infections diagnosed by a Rapid Diagnostic Test (RDT), including both symptomatic and asymptomatic cases. The RDT used in the study (SD bioline pf/pv combo test) can differentially detect HRP-II specific to *P. falciparum* and pLDH specific to *P. vivax*. The study involves both active and passive case detection. Effectively, incidence of *P. falciparum* and *P. vivax* infections will be compared using data between villages where personal insect repellent is being distributed and villages where it is not being distributed and between periods of non-repellent distribution and repellent distribution for the same villages.

The secondary research objective is to determine the impact of distributing personal repellent to villages through VHV on 1) the incidence of symptomatic malaria, defined as RDT positive plus fever and/or other malaria symptoms; 2) the incidence of PCR detectable *Plasmodium* spp. infections; 3) the prevalence of molecular markers of artemisinin resistance, and 4) antibody levels in individuals as determined by Enzyme Linked Immuno-sorbant assay (ELISA).

### Study setting and population

Malaria remains a leading cause of morbidity and mortality in Myanmar, which has the highest malaria burden in the GMS [12]. In 2015, there were an estimated 182,616 cases of malaria and 37 malaria deaths in Myanmar [13] and 22.5 million people (43% of the total population) reside in malaria endemic areas, with 21.4 million (41%) living in areas with receptivity and vulnerability risk of malaria [13]. Residence within 1 km of a forested area typically means that malaria transmission occurs in the village at least during part of the year, with all age groups at risk. If the village is further from the forest, risk is usually confined to adult men who enter the forest periodically to engage in agriculture, gathering forest produce, hunting, and other activities and to migrants seeking economic opportunities in rural areas [13]. According to data from 2012 to 2015, transmission is year-round with peak transmission in July and the lowest transmission in March and April [13]. In baseline studies, malaria incidence by RDT was estimated to be 1% (unpublished data).

The study sites represent isolated villages and hard-to-reach territories in the states of Kayin, Kayah and Bago (East) Region in South-east Myanmar identified by National Malaria Control Program (NMCP) data as lacking malaria health care services (Additional file 2). In these states malaria is most prevalent in rural and remote areas, particularly where mobile and internally displaced populations are living in conflict affected and/or ceasefire zones with little or no access to formal health care.

Burnet Institute Myanmar (BIMM) together with its local partner, Karuna Mission Social Solidarity (KMSS), in close consultation and partnership with the NMCP, initiated the artemisinin resistance containment and malaria control program in April 2014 with funding support from 3MDG (Three Millenium Development Goal Fund). BIMM and KMSS collaborate with local health authorities and the informal private health sector in Kayin and Kayah states and Bago (East) region to deliver malaria prevention (LLINs, repellent), early diagnosis (with RDTs), quality treatment (ACT, chloroquine and primiquine, for treatment regimes see Additional file 3, behaviour change and communication services (BCC) to local, mobile and internally displaced populations that are geographically and socially isolated. Program sites were selected in response to the malaria service gaps identified in NMCP services data, with priority areas being isolated and cut-off villages and hard to reach territories that lack adequate services. Prior to commencement of the study all villages had received LLINs either from BIMM or other Implementing Partners (IPs). The repellent intervention was intended to supplement, but not replace, currently used malaria treatment and prevention measures and in particular was aimed at addressing the additional vulnerability to infection faced by mobile and migrant populations: studies conducted in the region have suggested that migrant workers, particularly outdoor night-time workers, are less likely to sleep under LLIN than non-migrant workers [14, 15].

### Study design

The study is an open stepped-wedge cluster-randomised controlled trial, randomised at the village level. Using this approach repellent will be implemented sequentially across time in 116 villages, so that *all* villages ultimately receive the intervention during the observation period of the study. Implementation will be serviced by the 116 VHVs taking part in the 3MDG program. The stepped-wedge cluster randomised design involves rapid-diagnostic testing approximately 20 participants per month in each village between April 2015 and June 2016. Villages are grouped into blocks of 8 villages, with blocks transitioned monthly, with no transition period, from control (no repellent) to intervention states (receiving repellent) across 14 monthly intervals in random order (12 villages transitioned in the final month given the incomplete design) beginning in May 2015. This follows a one month baseline period in April 2015 where all villages do not receive repellent (Table 1).

**Table 1:** Study Schedule

TIMEPOINT (month)	Enrolment	Allocation	Post-allocation													
	*-t*_*2*_	*t*_*0*_	*t*_*1*_	*t*_*2*_	*t*_*3*_	*t*_*4*_	*t*_*5*_	*t*_*6*_	*t*_*7*_	*t*_*8*_	*t*_*9*_	*t*_*10*_	*t*_*11*_	*t*_*12*_	*t*_*13*_	*t*_*14*_
ENROLMENT:																
Eligibility screen^a^	X															
Community consent^b^		X	X	X	X	X	X	X	X	X	X	X	X	X	X	
Allocation		X														
REPELLENT INTERVENTION:																
*Block 1*		C	I	I	I	I	I	I	I	I	I	I	I	I	I	I
*Block 2*		C	C	I	I	I	I	I	I	I	I	I	I	I	I	I
*Block 3*		C	C	C	I	I	I	I	I	I	I	I	I	I	I	I
*Block 4*		C	C	C	C	I	I	I	I	I	I	I	I	I	I	I
*Block 5*		C	C	C	C	C	I	I	I	I	I	I	I	I	I	I
*Block 6*		C	C	C	C	C	C	I	I	I	I	I	I	I	I	I
*Block 7*		C	C	C	C	C	C	C	I	I	I	I	I	I	I	I
*Block 8*		C	C	C	C	C	C	C	C	I	I	I	I	I	I	I
*Block 9*		C	C	C	C	C	C	C	C	C	I	I	I	I	I	I
*Block 10*		C	C	C	C	C	C	C	C	C	C	I	I	I	I	I
*Block 11*		C	C	C	C	C	C	C	C	C	C	C	I	I	I	I
*Block 12*		C	C	C	C	C	C	C	C	C	C	C	C	I	I	I
*Block 13*		C	C	C	C	C	C	C	C	C	C	C	C	C	I	I
*Block 14*		C	C	C	C	C	C	C	C	C	C	C	C	C	C	I
ASSESSMENTS:																
*Rapid Diagnostic Test*		X	X	X	X	X	X	X	X	X	X	X	X	X	X	X
*Data Collection*		X	X	X	X	X	X	X	X	X	X	X	X	X	X	X
*Dried Blood Spot*		X	X	X	X	X	X	X	X	X	X	X	X	X	X	X
*Informed Consent*		X	X	X	X	X	X	X	X	X	X	X	X	X	X	X

*C* Control state, *I* Intervention state

^a^Screening of 3MDG villages for inclusion in the trial

^b^Community consent is gained one month prior to the distribution of repellent

All the VHV identified to take part in the trial receive repellent to distribute to villagers including mobile and migrant people and forest dwellers. There are several advantages to using a stepped-wedge cluster-randomised trial design over a parallel group design (where each cluster is randomised to either an intervention or control condition for the *entire* duration of the study). In the stepped-wedge design all clusters ultimately receive the intervention and this is important where the intervention is thought to have a largely positive effect and exclusion of study subjects from such benefit might be considered unethical. Also, clusters act as their own controls as they experience both the control and intervention conditions during the study period [16]; enabling analyses of any temporal effects of the intervention. Furthermore, given the repeated measurements, the effect of the intervention can be estimated using both between- and within-cluster information, and this can result in greater statistical power and effectively, smaller sample sizes compared to independent parallel group designs if the appropriate statisitically modelling is applied [16]. The study will be unblinded because participants and VHVs are actively participating in receiving and distributing the repellent intervention.

### Eligibility criteria

Villages included in the 3MDG malaria program, who had not previously received repellent, are considered for inclusion in this study. Individuals of all ages and genders willing to undergo RDT test for malaria are included in the analysis of the primary endpoint (*P. falciparum* and *P. vivax* infections) and the secondary endpoint of symptomatic malaria. Individuals of all ages and genders willing to provide additional biospecimens are included in the analysis of additional molecular and serological secondary endpoints. High risk populations (mobile and migrant people and residents who are also forest dwellers [17]) over 6 months of age (to ensure safe use of product) will receive the repellent. These groups are defined according to the guidelines on the prevention and control of malaria for migrants in Myanmar [17]. A migrant is defined as a person who takes up residence or remains in another place for an extended period of time, including seasonal migrants. A migrant moves from one location to another, regardless of duration or distance; experiences inequitable access to public health services resulting from the movement; and is vulnerable to becoming infected with malaria as a result of the movement [17]. Any person who is constantly moving, such as truck drivers, seafarers, travelling salespersons, “maw sayar”, and sex workers is defined as a mobile person [17]). Any person who regularly works in forests and stays overnight is defined as a forest dweller [17].

### Village recruitment and randomization

The study will recruit 116 project villages previously selected in response to malaria service gaps identified in NMCP services data (16 in Bago East; 61 in Kayin; 39 in Kayah state) (Additional file 2). All the villages identified to take part in the trial receive the repellent, but the time at which they can begin distribution is determined by random allocation (Table 1). Randomization of the timing of repellent distribution to villages is applied at the block level and is achieved by application of a block randomization routine written in the Stata statistical software package [18]. The randomization process is carried out by the statistician at the Burnet Institute, Melbourne, Australia with villages de-identified (using unique numeric identifiers) before randomization.

### Intervention

The type of insect repellent to be distributed is N, N-diethyl-benzamide – 12% w/w, cream. Clinical efficacy studies have shown that N,N-diethyl-benzamide – 12% w/w, cream is as efficacious as DEET (N,N-diethyl-3-methyl benzamide) when tested at a range of concentrations [11]. In human field studies N,N-diethyl-benzamide – 12% w/w, cream provides 100% protection against Anopheles mosquitoes and complete protection for up to 11 h [11]. N, N-diethyl-benzamide has been shown to be effective against both day biting and night biting mosquito species and is marketed locally for use against local mosquito species. No adverse reactions such as itching, irritation, vomiting, nausea etc., have been reported in human field trials [11]. The shelf life of N,N-diethyl-benzamide – 12% w/w, cream is 5 years (longer than the duration of this study). This repellent was chosen because it is widely available in Myanmar whereas DEET-containing products are not.

### Distribution of repellent

Repellent is distributed in plain tubes (unbranded) with appropriate instructions, warnings, manufacturing and expiration dates. Repellent is provided in unbranded tubes, to reduce the likelihood that recipients perceive the product itself as being promoted. Repellent is delivered to the village between one and two months prior to the onset of the rainy season and stored in an appropriate secure location prior to commencement of distribution to individuals.

Malaria officers (MO) of KMSS and VHVs deliver repellent to migrant and mobile workers and forest dwellers over six months of age (2 tubes/person) by visiting dwellings where they are staying, with MO distributing repellent the first time and VHV providing ongoing distribution. At the time of delivery, the nature of the study and proper usage of repellent is explained in ethnic language with the aid of the Guidelines for Repellent Usage (Additional file 4), and a paper copy of the Guidelines written in ethnic version (Myanmar, Karen and Kayaw) is provided to the repellent recipient.

Recipients of the repellent are told to inform their VHV before they run out of the product to enable its timely replacement and thus uninterrupted usage. In order to establish that repellent is being used, recipients are asked to return empty tubes and the number of tubes distributed to each recipient is recorded. In addition, as part of the 3MDG program VHV and village member surveys will capture data pertaining to implementation of the intervention, both in terms of repellent distribution at the village level and both VHV and village member assessment of the application, frequency of use and acceptability of repellent. VHV are responsible for ordering sufficient repellent from MO of KMSS to cover at least one month of stock in hand for all the beneficiaries within his/her reach. BIMM provides adequate stock to KMSS quarterly.

### Health education sessions

As part of the 3MDG artemisinin resistance containment and malaria control program, health information sessions are conducted by MO with the assistance of VHV in villages participating in the program. MO will inform the rural health centres and sub centres about the Health Education Sessions and invite designated basic health staff to participate. MO will inform local authorities (state/non-state) about the Health Education Session and request their participation in the session. Health Education Sessions include: an explanation of the 3MDG artemisinin resistance containment and malaria control program being implemented by BIMM and KMSS; Information about malaria, including its mode of transmission, signs and symptoms of malaria and danger signs and prevention strategies; an explanation of the free diagnosis and treatment service being offered by VHV. It is anticipated that village inhabitants will seek care for malaria from their local VHV for the following reasons: They do not have to travel to other locations; the VHV service is free; the quality of the treatment for uncomplicated malaria is comparable to that provided by basic health staff in the area; and VHV are trusted by village inhabitants because they are local residents themselves, who speak local dialect and understand the local context.

### Collection of endpoints

VHV and MO will collect data on *P. falciparum* and *P. vivax* infections in each village using both passive and active detection. Individuals presenting to VHV with fever (history of fever or temperature ≥ 37.5 °C) and/or malaria at risk behaviors will be tested for the presence of malaria parasites by RDT (SD bioline p.f / p.v combo test) (Passive case detection). VHV will perform RDT testing in mass screening programs with the help of MO in order to ensure all suspected cases (either symptomatic or asymptomatic) in the territory get parasitological confirmation for malaria (active case detection). VHV are educated about the possibility of HRP-2 and pLDH traces remaining in circulation post-treatment. If the patient returns for rapid diagnostic testing within one month after complete treatment, then they are referred for microscopy. High risk groups including mobile and migrant workers and forest dwellers will be identified by mapping all villages at the start of the project and this information will be updated yearly. The village mapping also serves to collect basic demographic data in the project villages. High risk groups will be targeted for active case detection. Individuals found to be parasite positive by RDT will be treated according to national guidelines and/or referred to nearest government health care facility according to the national guidelines. For each RDT administered by the VHV, information, including date, name, age group, address, sex, pregnancy status, RDT results, and treatment will be recorded on a “Village Health Volunteer Monthly Patient Record Form” which is the standard malaria collection tool for implementing partners created and used by the Myanmar NMCP (Additional file 5). Date, time, village, unique number, age and RDT result will also be recorded on the RDT to facilitate data validation. The forms will be forwarded to data assistants of KMSS, Township Health Department and State/ Regional Vector Borne Diseases Control office at the end of each month and data entered into a database by data assistants of KMSS. Both hard copies and electronic data will be transferred to BIMM for data compilation, double checking and processing. BIMM will then provide a clean database to BI Melbourne for analysis of primary and secondary outcomes.

### Collection of secondary endpoints

Individuals tested by RDT will be asked to provide another two drops of blood from their finger for collection on a piece of filter paper after informed consent. Filter papers are to be collected and stored in a sealed plastic bag containing silica gel to protect the samples from moisture. Each month, the filter papers will be collected by the Malaria Officers along with the Village Health Volunteer Monthly Patient Record Form and positive RDTs. The filter papers are to be sent to BI Melbourne for laboratory analysis. Filter papers form part of a Material Transfer Agreement (MTA) between the Myanmar Department of Public Health and the BI Melbourne, Australia.

Symptomatic malaria will be defined as a positive RDT in an individual reporting malaria symptoms (headache, lassitude, fatigue, abdominal discomfort and muscle and joint aches, usually followed by fever, chills, perspiration, anorexia, vomiting and worsening malaise [19]). PCR detectable *Plasmodium* spp. infection will be defined as detection of *P. falciparum, P. vivax, P. malariae,* and/or *P. ovale* parasite DNA by PCR. Prevalence and genetic structure of molecular markers of artemisinin resistance will be determined in PCR positive samples using standard established methods [20, 21]. Concentrations of antibodies to *Plasmodium* spp. antigens will be measured in filter paper samples by ELISA using standard established methods [22, 23].

### Statistical analysis

Quantitative data collected in the field will be entered into a database and subsequently imported into Stata for necessary data management and analysis [18]. Difference in risk of the primary outcome *P. falciparum* and *P. vivax* incidence will be estimated across intervention and control states using a generalised linear maximum likelihood mixed modelling (GLMM, a.k.a. multi-level modelling) analytical approach, with village clusters treated as a random effect and study group, time and seasonality estimated as independent fixed factors. Temporal and spatial trends of *P. falciparum* and *P. vivax* incidence will also be explored including analysis of the effectiveness of the intervention across the study period. Sandwich estimator variance estimation will be used to adjust for potential lack of independence in observations within clusters over the study period. Generalised linear mixed modelling will also be extended to facilitate modelling of both village and temporal (cross-sectional month) dependence underpinning *P. falciparum* and *P. vivax* infection incidence and the extent to which between-village heterogeneity is present in the effect of repellent distribution on incidence of these infections. Analyses of secondary outcomes will also involve multi-level modelling (i.e. linear mixed modelling (LLM) and GLMM). Analyses of the implementation will also be performed such as exploring rates of repellent uptake, reported side effects and adverse events (e.g. allergic reactions).

### Power estimation

Given the stepped-wedge design, expected study capacity for testing (20 tests per month per village) an estimate of malaria incidence of 1% (by RDT of both symptomatic and asymptomatic individuals, based on unpublished data from 3MDG malaria data collection in the three states during 2014), an estimate of between-village heterogeneity in incidence (ICC) of *ρ* = 0.15 and assuming repellent will reduce incidence of *P. falciparum* and *P. vivax* infections in an absolute sense to 0.5%, the study has sufficient power (> 90%) to detect an effect of this magnitude (~risk ratio 0.50) at the 5% significance level (two-sided). Power estimation was explicitly based on estimation of an intervention effect using trial data from a stepped-wedge cluster randomised design assuming analysis by GLMM [24].

### Data management

Personal information collected from participants will be limited to that entered into the Village Health Volunteer Monthly Patient record form (Additional file 5) and repellent distribution form. This information is routinely collected as part of the NMCP and investigators have permission to use these forms for research purposes. There will be no additional information sourced from medical records or participants for the study. BIMM will assign a unique identifier to all biospecimens (filter paper samples) and corresponding personal data. Only BIMM database administrators will have access to these unique identifiers but all unit-record data and samples used for research will be non-identifiable to data analysis staff and laboratory researchers at BI, Melbourne. The paper based data and consent forms will be kept separately in locked cupboards in BIMM and only assessible to the database administrators in BIMM. Unit-record data will be stored in an electronic database and on password protected secure Institute servers; accessible only those listed on the ethics submission. Data monitoring for this implementation trial will be performed by study investigators. Biological samples will be archived at the BI, Melbourne. Restrictions on the use of the data will be clearly recorded and kept with the original data sets. All principal investigators (both in Australia and host country) and trial statisticians will have access to the final trial data set.

### Ethical considerations

Ethical approval for the protocol has been obtained from the Ethics Review Committee on Medical Research involving Human Subjects, Department of Medical Research (21/Ethics/2015) and the Alfred Hospital, Melbourne, Australia (95/15). Good Clinical Practice training is provided to all staff/investigators prior to commencing the studies.

### Informed consent

The nature of the proposed research and the implications of participation in the study will be explained to village leaders and/or stakeholders in their language. Community consent for the distribution of insect repellent will be sought from village leaders or stakeholders (Additional file 4) but individuals will not be required to sign a consent form in order to receive repellent. An informed consent form written in ethnic language will be provided to men, women, and parents of children given a RDT for malaria (Additional file 6). The research study will be explained to prospective participants prior to rapid diagnostic testing. Whilst personal data relating to rapid diagnostic testing would otherwise be collected as part of the NMCP, the dried blood spots on filter paper represent a biospecimen that is to be collected solely for the purpose of research. If an individual chooses to provide a research biospecimen and signs the consent form, a finger prick sample of blood will be collected onto filter paper. Unrestricted informed consent will be requested for use of leftover filter paper samples in future studies. Any new tests not covered in the present protocol will not be carried out unless a separate approval is obtained from the relevant ethics committees in Myanmar and Melbourne.

### Risks and benefits

The risks from participation in the study are minimal. The finger-prick procedure for RDT administration and dried blood spot sample collection may be associated with minor discomfort or pain. The procedure for collecting blood for the filter paper sample is the same as for collecting blood for the RDT. Personal information collected from participants will be the same as that collected routinely by the NMCP. Whilst no adverse reactions such as itching, irritation, vomiting, nausea etc., have been reported in human field trials of the repellent [11], risks such as these, as well as accidental ingestion of the product, cannot be ruled out. Risks and benefits will be clearly explained in health information sessions and any questions raised by study participants will be addressed to ensure complete understanding prior to seeking informed consent. In the event that an adverse reaction to the repellent is reported to VHV, the repellent recipients will be advised to discontinue using the product. The client will be referred immediately to the nearest government health center for medical assessment and necessary treatment. Participants are assured that refusal to participate in the study will in no way affect access to health services in the community.

### Community engagement and dissemination

Community members, including the key stake holders (local authorities, non-state actors, health authorities) in the 3MDG program areas are aware of the malaria services of the program. The activities in the research will not involve community sensitive, socially, religiously and culturally inappropriate, and taboo procedures. Advocacy and coordination meetings for the research will be done through BIMM, KMSS National office and KMSS Diocesan Office leaders prior to the implementation of the research. In addition, the health education sessions to beneficiaries before the distribution of repellent and information to the clients before taking blood for RDT and filter paper are routine procedures and will augment community understanding of, and participation in, the research.

A final technical report will be produced and a final dissemination workshop will be held with the NMCP, Department of Medical Resarch (DMR) and other malaria implementing partners to disseminate the findings of the study. Findings may also be presented at international and national conferences and published in peer-reviewed journals.

## Discussion

Malaria epidemiology in the GMS exhibits great complexity and the disease is concentrated mainly in remote areas. The emergence of artemisinin resistance has increased the urgency of the malaria elimination agenda in the region. One of the key principles of the the GMS malaria elimination strategy is that all countries can accelerate efforts towards elimination through combinations of interventions tailored to local contexts. The key interventions proposed by the WHO GMS malaria elimination strategy are universal case detection and management and disease prevention in transmission areas, including vector control measures such as LLIN and IRS [4]. Topical insect repellent delivered by VHV represents a supplementary malaria control measure that may help to reduce residual malaria transmission, particularly in mobile and migrant workers who are potentially less protected by interventions like long lasting insecticide nets and indoor residual spraying.

Described herein is the protocol for a stepped-wedge cluster randomized controlled trial to determine the effectiveness of personal insect repellent (N,N-diethyl-benzamide – 12% w/w, cream) distributed by VHV in reducing the incidence of *P. falciparum* and *P. vivax* infections in geographically isolated, high risk populations in Myanmar. Secondary outcomes include the impact of the intervention on molecular-detected *P. falciparum* and *P. vivax* parasites, parasite mutations that confer drug resistance, and on naturally-acquired immunity to malaria in the targeted populations. The intervention is randomised at the village level so that repellent is implemented sequentially across time in 116 villages. The key advantage of this stepped-wedge cluster-randomised controlled trial design is that all villages ultimately receive the intervention during the observation period of the study as well as the relatively large number of measurements of *Plasmodium* spp. infection taken across time which enables analysis of temporal trends for each of the primary and secondary outcomes. Morever, to our knowledge this is the first trial of the effectiveness of repellent in reducing the incidence of *P. falciparum* and *P. vivax* infection which uses a stepped-wedged study design and the first which also proposes use of relatively sophisticated multi-level modelling to appropropriately account for and estimate village and temporal heterogeneity in *P. falciparum* and *P. vivax* incidence while determining effectiveness.

The proposed study will provide an evidence base for the use of repellents in malaria control and elimination programs in the GMS and findings will contribute to a better understanding of the optimal distribution mechanisms of repellent. The stepped-wedge cluster randomized controlled trial design may prove useful for testing other malaria interventions in the context of large-scale artemisinin resistance containment and malaria control programs in the GMS, and disease prevention programs more broadly.

## Additional files

Additional file 1: SPIRIT checklist. (DOC 241 kb)

Additional file 2: Villages to be included in 3MDG-funded malaria project. (DOCX 142 kb)

Additional file 3: Treatment regimes. (DOCX 20 kb)

Additional file 4: Guidelines for Repellent Usage. (DOCX 888 kb)

Additional file 5: Village Health Volunteer Monthly Patient record form (English translation). (DOCX 180 kb)

Additional file 6: Community and individual consent forms for study (English translation). (DOCX 61 kb)

## Abbreviations

3MDG: Three Millennium Development Goal (3MDG) Fund
ACD: Active Case Detection
ACT: Artemisinin based Combination Therapy
BCC: Behavioural Change Communication
BHS: Basic Health Staff
BI: Burnet Institute
BIMM: Burnet Institute Myanmar
DMR: Department of Medical Research
ELISA: Enzyme Linked Immuno-sorbant assay
GLMM: Generalised Linear Mixed Modelling
GMS: Greater Mekong Sub-Region
ICC: Intraclass Correlation Coefficent
IP: Implementing Partner
IRS: Indoor Residual Spraying
KMSS: Karuna Mission Social Solidarity
LLIN: Long Lasting Insecticidal Net
LMM: Linear Mixed Modelling
MARC: Myanmar Artemisinin Resistance Containment
MO: Malaria Officer (KMSS)
NMCP: National Malaria Control Program
PCD: Passive Case Detection
PCR: Polymerase Chain Reaction
RDT: Rapid Diagnostic Test
SEA: South East Asia
SPIRIT: Standard Protocol Items: Recommendations for Interventional Trials
VHV: Village Health Volunteer
WHO: World Health Organization
