# Supplementary material for: Effectiveness of repellent delivered through village health volunteers on malaria incidence in villages in South-East Myanmar: a stepped-wedge cluster-randomised controlled trial protocol
Source: BMC Infect Dis. 2018 Dec 14;18:663. doi: 10.1186/s12879-018-3566-y (PMC6295052; doi:10.1186/s12879-018-3566-y)
Supplement: Supplementary file 2 — Villages to be included in 3MDG-funded malaria project. (DOCX 142 kb) [file 12879_2018_3566_MOESM2_ESM.docx]

Villages to be included in 3MDG-funded malaria project

| **No.** | **State** | **Township** | **Village tract** | **Village/Workplace** | **Household** | **Population** | **Adult males >5** |
| --- | --- | --- | --- | --- | --- | --- | --- |
| 1 | Bago (East) | Shwe Kyin | Le War | Baw Ka Htar | 177 | 838 | 368 |
| 2 | Bago (East) | Shwe Kyin | Kan Bay Aing | Yae Arr (Lote Thar Ywar) | 272 | 727 | 315 |
| 3 | Bago (East) | Shwe Kyin | Pa De Kaw | Shu Khin Thar | 58 | 262 | 124 |
| 4 | Bago (East) | Shwe Kyin | Pa De Kaw | Pa De Kaw | 140 | 689 | 321 |
| 5 | Bago (East) | Kyauk Ta Gar | Nyaung Pin Thar | Kyee Ma Noe | 30 | 120 | 46 |
| 6 | Bago (East) | Kyauk Ta Gar | Sar Pyin | Ywar Haung | 98 | 205 | 86 |
| 7 | Bago (East) | Kyauk Ta Gar | Myo Chaung | Five Mile | 30 | 150 | 52 |
| 8 | Bago (East) | Nyaunglebin | Swel Tha Lel | Min Ga Lar Kone | 94 | 472 | 188 |
| 9 | Bago (East) | Nyaunglebin | Inn Wein | Yae Kyi Chaung | 110 | 770 | 309 |
| 10 | Bago (East) | Kyauk Ta Gar | Myo Chaung | Nyaung Pin Sin |  |  |  |
| 11 | Bago (East) | Nyaunglebin | Kyan Bo | Nyaung Pin Thar | 190 | 887 | 410 |
| 12 | Bago (East) | Nyaunglebin | Zee Kone Gyi | Shan Su | 103 | 508 | 238 |
| 13 | Bago (East) | Nyaunglebin | Thaung Gyi | Pyi Ko Taw | 259 | 829 | 191 |
| 14 | Bago (East) | Nyaunglebin | War Yon Kone | La Thar Kone | 164 | 723 | 336 |
| 15 | Bago (East) | Nyaunglebin | War Yon Kone | Taung Thu Kone | 45 | 203 | 87 |
| 16 | Bago (East) | Nyaunglebin | Chaung Yin | Sin Ku | 198 | 1072 | 460 |
| 17 | Kayin | Leik Tho | Ah Doe Thea Pyaw (Leik Tho Sub-township) | Thea Pyaw (Lower) | 38 | 526 | 213 |
| 18 | Kayin | Leik Tho | Ah Doe Thea Pyaw (Leik Tho Sub-township) | A Doe Thae Pyaw (KDP) | 39 | 201 | 78 |
| 19 | Kayin | Leik Tho | Kyay Min (Leik Tho Sub-township) | Kyay Min (Lower) | 63 | 339 | 149 |
| 20 | Kayin | Leik Tho | Kyay Min (Leik Tho Sub-township) | Kyay Min (Ywar Thit) | 23 | 83 | 30 |
| 21 | Kayin | Leik Tho | Hnget Pyaw Taw (Leik Tho Sub-township) | Nar Pa Law Khaw | 23 | 105 | 40 |
| 22 | Kayin | Thandaunggyi | Leik Pyar Ka Lay | Leik Pyar Ka Lay (Lower) | 13 | 63 | 27 |
| 23 | Kayin | Thandaunggyi | Leik Pyar Ka Lay | Leik Pyar Ka Lay (Upper) | 14 | 56 | 20 |
| 24 | Kayin | Thandaunggyi | Leik Pyar Ka Lay | Mg Nwel Ka Lay Ywar Haung | 18 | 78 | 36 |
| 25 | Kayin | Thandaunggyi | Leik Pyar Ka Lay | Mg Nwel Ka Lay Ywar Haung | 15 | 85 | 31 |
| 26 | Kayin | Thandaunggyi | Khon Taing | La Mae Kyi (Khaung Law Kar) | 41 | 175 | 77 |
| 27 | Kayin | Thandaunggyi | Khon Taing | La Me Ka Lae | 34 | 190 | 70 |
| 28 | Kayin | Thandaunggyi | Kywe Hpyu Taung | Taw Pyar Ka Lay | 68 | 302 | 144 |
| 29 | Kayin | Thandaunggyi | Kywe Hpyu Taung | Yit Maing | 47 | 304 | 123 |
| 30 | Kayin | Leik Tho | Leik Tho Gyi (Leik Tho Sub-township) | Mar Day | 48 | 187 | 68 |
| 31 | Kayin | Leik Tho | Leik Tho Gyi (Leik Tho Sub-township) | Kyaung Kone (Leik Tho Kyaung Kone) | 99 | 535 | 193 |
| 32 | Kayin | Leik Tho | Za Le (Leik Tho Sub-township) | Lan Ku | 73 | 475 | 160 |
| 33 | Kayin | Leik Tho | Thar Moe Taung (Leik Tho Sub-township) | Thit War Taw | 18 | 103 | 48 |
| 34 | Kayin | Leik Tho | Thar Moe Taung (Leik Tho Sub-township) | Hei Moe | 11 | 77 | 26 |
| 35 | Kayin | Leik Tho | Hta Mon (Leik Tho Sub-township) | Hpar Tee Mar Kone | 34 | 168 | 76 |
| 36 | Kayin | Leik Tho | Hta Mon (Leik Tho Sub-township) | Boe Ka Lay | 62 | 344 | 155 |
| 37 | Kayin | Leik Tho | Min Lan Taung (Leik Tho Sub-township) | Kywe Kya (Up) | 35 | 177 | 53 |
| 38 | Kayin | Leik Tho | Min Lan Taung (Leik Tho Sub-township) | Kywe Kya (Lower) | 73 | 375 | 160 |
| 39 | Kayin | Leik Tho | Min Lan Taung (Leik Tho Sub-township) | Kywe Kya (Lower-Ywar Thit)/ Aung Za Li | 18 | 78 | 35 |
| 40 | Kayin | Leik Tho | Kyauk Gyi Taung (Leik Tho Sub-township) | Kyauk Gyi Taung (Ywar Thit) | 74 | 234 | 108 |
| 41 | Kayin | Leik Tho | Ka Lay Kho (Leik Tho Sub-township) | Taung Chaung (Lower) |  | 66 | 30 |
| 42 | Kayin | Leik Tho | Ka Lay Kho (Leik Tho Sub-township) | Taung Chaung (Middle) | 43 | 220 | 91 |
| 43 | Kayin | Leik Tho | Maung Kyaw (Leik Tho Sub-township) | Maung Kyaw (Upper) |  | 0 |  |
| 44 | Kayin | Leik Tho | Htee Thar Saw (Leik Tho Sub-township) | Nar Law Hpo Li (Lower) | 47 | 363 | 167 |
| 45 | Kayin | Leik Tho | Htee Thar Saw (Leik Tho Sub-township) | Nar Law Hpo Li (Lower Ywar Thit) | 16 | 102 | 45 |
| 46 | Kayin | Leik Tho | Dar Yoe (Leik Tho Sub-township) | Khay Chee | 45 | 239 | 104 |
| 47 | Kayin | Leik Tho | Htee Thar Saw (Leik Tho Sub-township) | Nar Law Hpo Li (Middle) | 14 | 98 | 43 |
| 48 | Kayin | Leik Tho | Ho Thaw Pa Lo (Leik Tho Sub-township) | Mei Thaw Hpo Li | 30 | 180 | 71 |
| 49 | Kayin | Leik Tho | Ho Thaw Pa Lo (Leik Tho Sub-township) | Khaw Thaw Khaw | 22 | 109 | 23 |
| 50 | Kayin | Leik Tho | Kha Mar Di Hpo Li (Leik Tho Sub-township) | Maw Chee | 37 | 192 | 93 |
| 51 | Kayin | Leik Tho | Kyauk Gyi Taung (Leik Tho Sub-township) | Ma Gyi Taw | 15 | 67 | 35 |
| 52 | Kayin | Leik Tho | Chee Thu Saw (Lower) (Leik Tho Sub-township) | Chee Thu Saw (Lower Ywar Thit) | 38 | 184 | 73 |
| 53 | Kayin | Leik Tho | Kha Mar Di Hpo Li (Leik Tho Sub-township) | Mar Yaw Hpo Li | 45 | 260 | 54 |
| 54 | Kayin | Leik Tho | Kha Mar Di Hpo Li (Leik Tho Sub-township) | Mar Hsar Khaw | 37 | 190 | 75 |
| 55 | Kayin | Leik Tho | Hnget Pyaw Taw (Leik Tho Sub-township) | Shwe Nan Ka Lay | 60 | 330 | 140 |
| 56 | Kayin | Leik Tho | Maing Lun (Leik Tho Sub-township) | Maing Lun (Lower) | 79 | 426 | 181 |
| 57 | Kayah | Hpruso | Rar Aye Pa Rar | Khaw Bar | 42 | 22 |  |
| 58 | Kayah | Hpruso | Do Mo Saw | Khaw Tha Khaw | 32 | 170 | 76 |
| 59 | Kayah | Hpruso | Do Mo Saw | Do Mo Saw | 27 |  |  |
| 60 | Kayah | Hpruso | Do Mo Saw | Yaw Du / Khaw Tha Khaw (Ywar Thit) | 21 | 132 | 50 |
| 61 | Kayah | Hpruso | Do Mo Saw | Khar Bei | 70 | 350 | 154 |
| 62 | Kayah | Hpruso | Ho Yar | Htay Kho | 75 | 350 | 150 |
| 63 | Kayah | Hpruso | Kay Kaw | Hpar Wei | 67 |  |  |
| 64 | Kayah | Hpruso | Kay Kaw | Saw Lel @ Khaw Taw Kho | 29 | 158 | 61 |
| 65 | Kayah | Hpruso | Kay Kaw | Bwei Do Thar | 32 |  |  |
| 66 | Kayah | Hpruso | Kay Kaw | Saw Pa Lay Kho @ Lu Dae | 28 | 128 | 45 |
| 67 | Kayah | Hpruso | Kay Kaw | Kay Kaw | 26 | 137 | 64 |
| 68 | Kayah | Hpruso | Mo So | Ku Khu | 20 | 117 | 45 |
| 69 | Kayah | Hpruso | Doe Lar Saw | Yo Pa Yar | 26 | 170 | 85 |
| 70 | Kayah | Hpruso | Doe Lar Saw | Doe Lar Saw | 35 | 170 | 70 |
| 71 | Kayah | Hpruso | Kay Kaw | Yo Li Kho | 29 | 130 | 50 |
| 72 | Kayah | Hpruso | Doe Lar Saw | Hto Khwi So | 94 | 480 | 195 |
| 73 | Kayah | Hpruso | Rar Aye Pa Rar | Rar Aye Pa Rar | 52 | 181 |  |
| 74 | Kayah | Hpruso | Rar Aye Pa Rar | Yo Sa Pa Yar | 90 | 386 |  |
| 75 | Kayah | Hpruso | Mo So | Maw Thi Do | 26 | 130 | 51 |
| 76 | Kayah | Hpruso | Kay Kaw | Khaw Lay | 21 | 108 | 47 |
| 77 | Kayah | Hpruso | Raw Daw Khaw | Raw Daw Khaw | 82 | 350 | 140 |
| 78 | Kayah | Hpruso | Raw Daw Khaw | Du Kho | 15 | 83 | 31 |
| 79 | Kayah | Hpruso | Raw Daw Khaw | Si Mi Dae/ Bay Bo Khaw | 24 | 97 | 36 |
| 80 | Kayah | Hpruso | Kay Kaw | Htaw Tho Khaw | 37 | 195 | 70 |
| 81 | Kayah | Hpruso | Tho Thee Hpo | Tho Thee Hpo | 24 | 151 | 66 |
| 82 | Kayah | Hpruso | Tho Thee Hpo | Doe Mu Khaw | 19 | 98 | 38 |
| 83 | Kayah | Hpruso | Kay Kaw | Htee War Khaw | 38 | 180 | 70 |
| 84 | Kayah | Hpruso | Tho Thee Hpo | Pa Yel Saw Khu | 14 | 65 | 25 |
| 85 | Kayah | Hpruso | Raw Daw Khaw | Htar Du Khee | 26 | 136 | 52 |
| 86 | Kayah | Hpruso | Kay Kaw | Hpaw Law | 42 | 178 | 75 |
| 87 | Kayin | Hpapun | War Kaw Kyay (Kamamaung Sub-township) | Ta Dar U | 155 | 769 | 290 |
| 88 | Kayin | Hpa-An | Hat Ta Laik | Yae Win | 11 | 50 | 22 |
| 89 | Kayin | Hpa-An | Me Tha Na | Me Tha Na Ywar Lay (1) | 44 | 264 | 90 |
| 90 | Kayin | Hpa-An | Me Tha Na | Me Tha Na Ywar Lay (2)* |  |  |  |
| 91 | Kayin | Hpa-An | Me Tha Na | Htee Kyu | 35 | 148 | 60 |
| 92 | Kayin | Hpa-An | Hat Ta Laik | Htee Hpae Do Khee (1) | 40 | 216 | 85 |
| 93 | Kayin | Hpa-An | Hat Ta Laik | Htee Hpae Do Khee (2)* |  |  |  |
| 94 | Kayin | Hpa-An | Hat Ta Laik | Noe Khar Day | 36 | 191 | 70 |
| 95 | Kayin | Hpa-An | Hat Ta Laik | Htee Kyaw Khee | 40 | 172 | 56 |
| 96 | Kayin | Hpa-An | Hat Ta Laik | Me Tha Na (1) | 44 | 138 | 22 |
| 97 | Kayin | Hpa-An | Hat Ta Laik | Me Tha Na (2)* |  |  |  |
| 98 | Kayin | Hpa-An | Mee Zaing | Bar Baung Ywar Lay | 30 | 156 | 70 |
| 99 | Kayin | Hpa-An | Mee Zaing | Ka Kyaw | 15 | 112 | 50 |
| 100 | Kayin | Hpa-An | Mee Zaing | Noe Ka Baw | 20 |  |  |
| 101 | Kayin | Hpa-An | Pyin Ma Pin Seik | Ta Kay Laung Chaung Phyar | 35 | 100 | 40 |
| 102 | Kayin | Hpa-An | Kawt Yin (Lower) | Ah Nyaw Kone | 30 | 100 | 37 |
| 103 | Kayin | Hpa-An | Kawt Yin (Lower) | Kawt Kareik Kone | 28 | 98 | 29 |
| 104 | Kayin | Hpa-An | Kawt Yin (Lower) | Shwae U Daun | 43 | 222 | 80 |
| 105 | Kayin | Hpapun | Nan Khu Khee | Htee Wal Hta | 78 | 475 | 208 |
| 106 | Kayin | Hpapun | Min Nan Nwe | War Mi Day | 46 | 270 | 104 |
| 107 | Kayin | Hpapun | Min Nan Nwe | Ka Law U Doe | 62 | 480 | 195 |
| 108 | Kayah | Demoso | Daw Yauk Khu | Le Ma An Khu Ywar Haung | 50 | 254 | 115 |
| 109 | Kayah | Demoso | Daw Yauk Khu | Ni Du Khu | 13 | 78 | 21 |
| 110 | Kayah | Demoso | Daw Yauk Khu | Hpa Ru Khaw | 34 | 187 | 74 |
| 111 | Kayah | Demoso | Daw Yauk Khu | Daw Yauk Khu | 241 | 1467 | 737 |
| 112 | Kayah | Demoso | Daw Yauk Khu | Han Li | 57 | 302 | 115 |
| 113 | Kayah | Demoso | Daw Yauk Khu | Daw Wel Khu | 42 | 300 | 135 |
| 114 | Kayah | Demoso | Daw Yauk Khu | Khu Bar To | 44 | 289 | 143 |
| 115 | Kayah | Demoso | Daw Yauk Khu | Bar To | 19 | 88 | 36 |
| 116 | Kayah | Demoso | Daw Yauk Khu | Si Saw | 14 | 91 | 43 |

*Denotes villages listed twice because they have more than one VHV. There are a total of 116 VHV representing 116 village/workplaces.
