# Supplementary material for: Effectiveness of repellent delivered through village health volunteers on malaria incidence in villages in South-East Myanmar: a stepped-wedge cluster-randomised controlled trial protocol
Source: BMC Infect Dis. 2018 Dec 14;18:663. doi: 10.1186/s12879-018-3566-y (PMC6295052; doi:10.1186/s12879-018-3566-y)
Supplement: Supplementary file 3 — Treatment regimes. (DOCX 20 kb) [file 12879_2018_3566_MOESM3_ESM.docx]

# Summary of Malaria Treatment Regimes administered by Village Health Volunteers:

**Treatment of uncomplicated P.f malaria ( P.f alone)**

Artesunate + Mefloquine x 3 days plus Primaquine 0.75 mg/kg stat dose on day 0.

Artemether + Lumefantrine x 3 days plus Primaquine 0.75 mg/kg stat dose on day 0 (NMCP adopted combination)

Dihydroartemisinin + piperaquine x 3 days plus Primaquine 0.75 mg/kg stat dose on day 0

**Treatment of uncomplicated mix infection (P.f + non-P.f)**

Artesunate + Mefloquine x 3 days plus Primaquine 0.75 mg/kg/week x 8 weeks.

Artemether + Lumefantrine x 3 days plus Primaquine 0.75 mg/kg/week x 8 weeks (NMCP adopted combination)

Dihydroartemisinin + piperaquine x 3 days plus Primaquine 0.75 mg/kg/week x 8 weeks

**Treatment of uncomplicated Non-P.f malaria (P.v, P.o or P.m)**

Chloroquine 25 mg base/ kg on day 0, 1 and 2 plus Primaquine 0.75 mg/kg/week x 8 weeks
