# Supplementary figures and images for: Effectiveness of repellent delivered through village health volunteers on malaria incidence in villages in South-East Myanmar: a stepped-wedge cluster-randomised controlled trial protocol
Source: BMC Infect Dis. 2018 Dec 14;18:663. doi: 10.1186/s12879-018-3566-y (PMC6295052; doi:10.1186/s12879-018-3566-y)

Guidelines for Repellent Usage


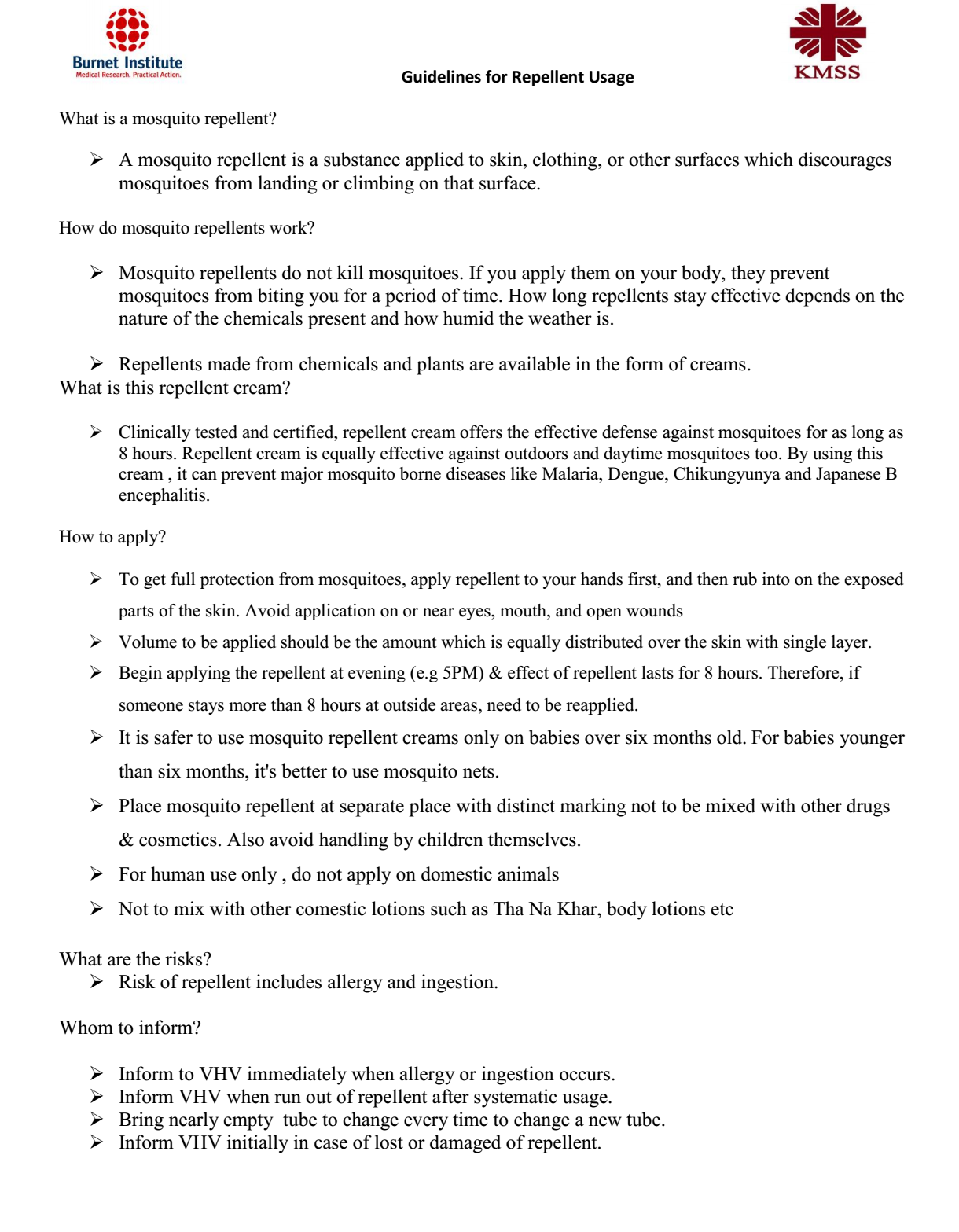

Supplement: Supplementary file 4 — Guidelines for Repellent Usage. (DOCX 888 kb) [file 12879_2018_3566_MOESM4_ESM.docx]

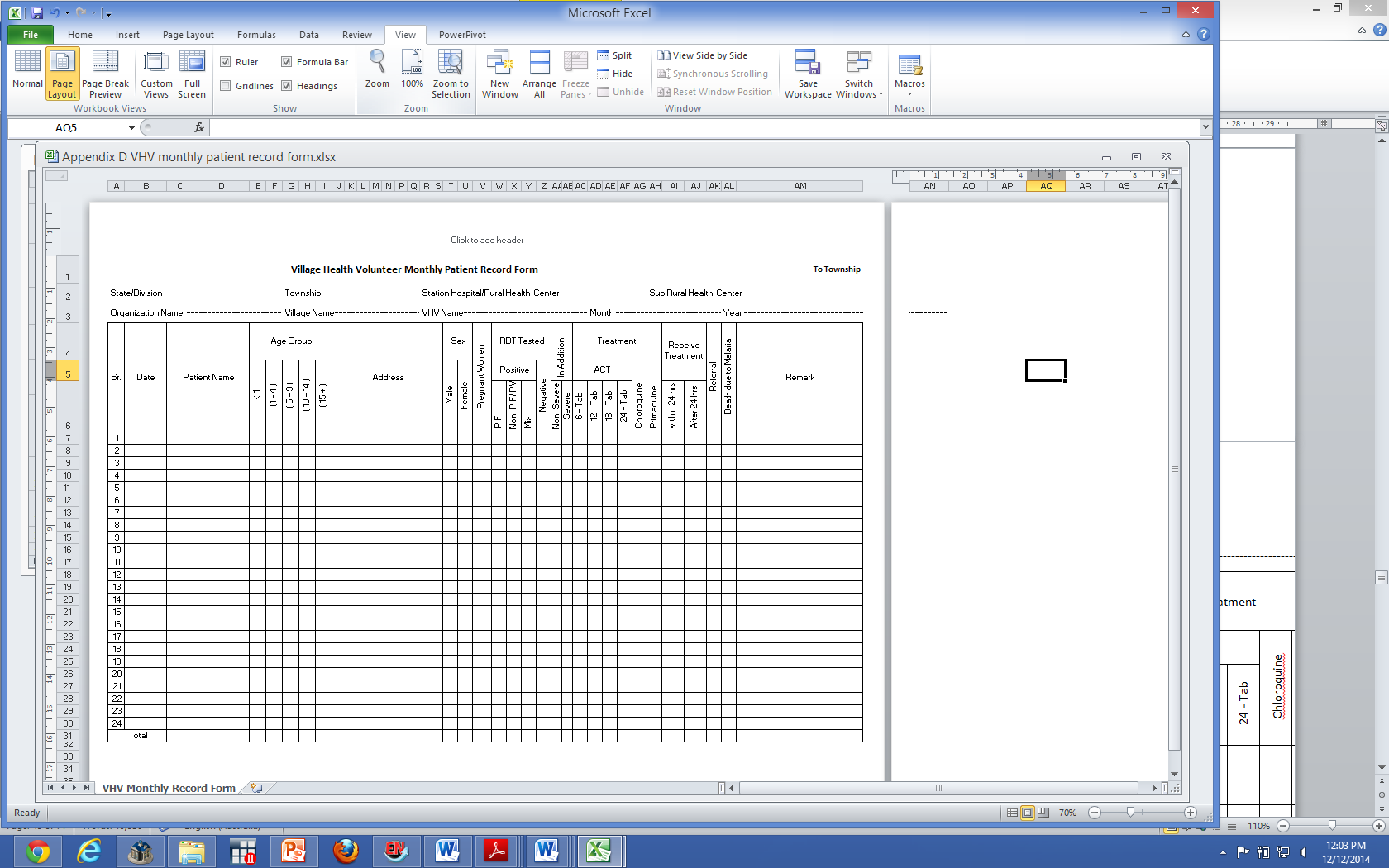


Signature…………………………………….

Name………………………………………….

Village…………………………………………

Organization……………………………….

Supplement: Supplementary file 5 — Village Health Volunteer Monthly Patient record form (English translation). (DOCX 180 kb) [file 12879_2018_3566_MOESM5_ESM.docx]
