# Supplementary material for: Effectiveness of repellent delivered through village health volunteers on malaria incidence in villages in South-East Myanmar: a stepped-wedge cluster-randomised controlled trial protocol
Source: BMC Infect Dis. 2018 Dec 14;18:663. doi: 10.1186/s12879-018-3566-y (PMC6295052; doi:10.1186/s12879-018-3566-y)
Supplement: Supplementary file 6 — Community and individual consent forms for study (English translation). (DOCX 61 kb) [file 12879_2018_3566_MOESM6_ESM.docx]

**Ethics Review Committee**

**Department of Medical Research**

**(Lower Myanmar)**

**Yangon, Myanmar**

**Community Consent Form for a study of the effectiveness of repellent in preventing malaria**

This community consent form is for leaders or key stakeholders of villages and worksites that are part of the Malaria Control Program of Burnet Institute in South-East Myanmar. We are inviting individuals to participate in a study looking at the effectiveness of personal insect repellent in preventing malaria and stopping the spread of drug resistance. This community consent form will be given to leaders or key stakeholders to seek consent for the timed distribution of personal insect repellent to high risk groups, including mobile and migrant workers and forest dwellers living in their community.

**PART I: Information Sheet**

**Introduction**

We are working for the Burnet Institute and the National Malaria Control Program and we are doing research on malaria in Myanmar. As a leader or key stakeholder of a village or worksite, we are going to give you information and invite your village/workplace to be part of this research. Before you decide whether you would like your village/workplace to participate, you can talk to anyone you feel comfortable with about the research. There may be some words that you do not understand. Please ask us to stop as we go through the information and we will take time to explain. If you have questions later, you can ask them of your Malaria Officer.

**Purpose**

Malaria is a common cause of illness in Myanmar and is spread by mosquito bites. In Myanmar, malaria is becoming resistant to drugs and unfortunately this resistance is spreading. One way to prevent getting malaria and to stop the spread of drug resistance is by using insect repellent, but we want to know how effective this will be in South-East Myanmar. We would like to distribute personal insect repellent to mobile and migrant workers and forest dwellers and measure whether it helps to prevent them and others in your community from getting malaria.

**Type of Research Intervention**

If you agree to allow your village/workplace to participate, we will provide personal insect repellent to mobile and migrant workers and forest dwellers in your village/workplace at a specific date, decided in advance by the research team. Village Health Volunteers and Malaria Officers will distribute the repellent to mobile and migrant workers and forest dwellers and explain the proper usage of repellent.

In order to measure whether the repellent helps prevent malaria in your village/workplace, we will collect information about malaria rapid diagnostic tests conducted in your village. When an individual in your village/workplace comes for a rapid diagnostic test, we will ask them to participate in this study. If they agree to participate, we will collect some information about them and about the test.

Immediately after the rapid diagnostic test, we will ask the person to provide another small sample of blood (two drops) from their finger that will be collected on a piece of filter paper. We will send this filter paper to a laboratory where we will test to see whether there are signs that the person has had malaria recently. We will also use the blood to see whether it contains malaria parasite material. We will test the parasite material to see whether the parasite is likely to be resistant to a malaria drug called artemisinin.

**Participant selection**

This study includes villages and workplaces that are taking part in the National Malaria Control Program as well as the Malaria Control Program of Burnet Institute. Mobile and migrant workers, as well as forest dwellers will be selected to receive insect repellent because they generally have a higher risk of malaria than people who live in the village permanently. Mapping of migrant and mobile worker populations and forest dwellers will be conducted prior to distribution of repellent to identify where they are staying. As well as providing repellent to mobile and migrant workers and forest dwellers, we are inviting everyone who is having a malaria rapid diagnostic test to participate in this study. If an individual participates in this study we will collect some information about them and the malaria test and we will take an additional two drops of blood for research.

**Voluntary Participation**

It is your choice whether your village will participate in this research study or not. Whether you choose for your village to participate or not will not change access to malaria testing or malaria treatment in your village. Individuals in your village are free to change their minds later and stop participating in the study, even if they agreed earlier.

**Procedures and Protocol**

In this study we will provide personal insect repellent to mobile and migrant workers and forest dwellers. The date when insect repellent will be distributed in your village will be decided in advance by the research team using computerized random allocation. Village Health Volunteers and Malaria Officers will distribute the repellent to mobile and migrant workers and forest dwellers. At the time of delivery, correct usage of repellent will be explained and a repellent distribution form will be completed to record who has received repellent. Individuals provided with insect repellent will be told to inform their Village Health Volunteer before they run out of repellent, and to bring the empty tube which will be replenished to continue the usage of repellent.

When an individual comes for a rapid diagnostic test to see whether they have malaria, it is routine procedure for the government to collect information about the test to help show how much malaria there is in the village. We will use this routine information to study whether insect repellent helps prevent malaria in your village/workplace. We will also ask individuals to give another two drops of blood onto a piece of filter paper after their finger has been pricked for the rapid diagnostic test. The blood on the filter paper will be sent to a laboratory in Australia to see whether there are signs the person has had malaria recently. We will also look at any malaria parasite material that might be in the person’s blood to see whether the parasite is resistant to the malaria drug artemisinin.

The blood on the filter paper that we take from individuals will be stored and may be used for further studies. We will provide individual consent forms to people when they present for rapid diagnostic testing asking permission to collect information about the rapid diagnostic test and to take a finger prick blood sample.

**Duration**

This study will run 45 minutes or 1 hour, but there will be no follow-up of individual participants. This means that after an individual has had a rapid diagnostic test and provided a finger prick sample of blood on filter paper, they will not need to do anything else to be part of the study. If at a later date if they are given another rapid diagnostic test they may be asked to participate in the study again. The repellent will be distributed to the same individuals in your community up to the end of research, October 2016 and we will not come to you again for consent unless there are changes in the research and information provided in this consent form.

**Risks and discomfort**

The risks of participation in the study are minimal. Upon delivery, correct usage of insect repellent will be explained to recipients to reduce the likelihood that it is applied inappropriately. The finger-prick procedure for RDT administration and blood sample collection may be associated with minor discomfort or pain. The procedure for collecting blood for the filter paper sample is the same as for collecting blood for the RDT. In some cases, a second finger prick will be taken. Personal information collected from participants will be the same as that collected routinely by the National Malaria Control Program. Risk of repellent includes allergy and ingestion.

**Benefits**

There may be no benefit to individuals in the village, but participation in this study may help us understand more about malaria in South-East Myanmar. In particular, your village’s involvement in this study can help us work out whether insect repellent can help prevent malaria in this region and help stop the spread of drug resistance.

**Incentives**

Individuals will not be given any money or gifts to take part in this study.

**Confidentiality**

The information that we collect from this research project will be kept confidential and no one but the researchers will be able to see it. Any information about individuals will have a number on it instead of a name. Only the researchers will know what an individual’s number is and we will keep it safely.

**Sharing the Results**

The knowledge that we get from doing this research will be reported back to your village/workplace and Village Health Volunteers before it is made widely available to the public. We will publish the results and may share the results at conferences so that other people may learn from our research. Confidential information will not be shared.

**Right to Refuse or Withdraw**

Individuals may stop participating in the research at any time without losing their right to malaria testing and treatment. Testing or treatment for malaria in the village will not be affected if you decide that your village will not participate in the study.

**Who to Contact**

If you have any questions you may ask them now or later, even after the study has started. If you wish to ask questions later, you may contact any of the following people:

Dr Win Han Oo

Phone number: 01- 375785, 375763, 375727 Ext 305 or 09 3190 6318

Burnet Institute Myanmar, No. 226, 2nd Floor, Wizaya Plaza, U Wisara Road, Bahan Township,11201 Yangon, Myanmar

This proposall will be reviewed by the Ethics Review Committee, Department of Medical Research (Lower Myanmar) which is a committee whose task is to make sure that research participants are protected from harm. If you wish to find out more about the Committee, contact the secretary of the committee at the Department of Medical Research (Lower Myanmar), No 5 Ziwaka Road, Dagon PO, Yangon, phone 375457- ext: 118.

In addition, this proposal will be reviewed by the Alfred Health Human Ethics Committee, Office of Ethics & Research Governance, Ground Floor, Linay Pavilion, The Alfred,
55 Commercial Rd, Melbourne VIC 3004, Australia.

**PART II: Certificate of Consent**

My village/workplace has been invited to participate in a study of the effectiveness of repellent in preventing malaria. I understand that it will involve the provision of personal insect repellent to mobile and migrant workers and forest dwellers at a specified date. In addition, individuals having rapid diagnostic tests for malaria will be asked to participate in the study by allowing us to collect information about the test and by providing a small finger prick sample of blood to be collected onto a piece of filter paper. Individuals receiving rapid diagnostic tests will be given informed consent forms to complete to provide consent for this part of the study. I have been informed that the risks of participation in the study are minimal and may include only minor discomfort associated with taking drops of blood from the finger with a lancet. I am aware that there may be no benefit to myself or to individuals in the village and that we will not be given money or gifts for participating in the study. I have been provided with the name of a researcher and appropriate persons who can be contacted using the number and address I was given for that person.

***I have read the foregoing information, or it has been read to me. I have had the opportunity to ask questions about it and any questions that I have asked have been answered to my satisfaction. As leader or key stakeholder I consent to my village participating in this research and understand that individuals have the right to withdraw from the research at any time without in any way affecting their medical care.***

**Name of leader or key stakeholder_______________________**

**Signature of leader or key stakeholder___________________**

**Date ____________________________________**

**Day/month/year**

***If illiterate***

A literate **witness must sign** (if possible, this person **should be selected by the village leader** and should have **no connection to the research team**). Village leaders who are illiterate should include their **thumb-print** as well.

**Thumb print of leader or key stakeholder:**

***I have witnessed the accurate reading of the consent form to the village leader, and the***

***individual has had the opportunity to ask questions. I confirm that the individual has given consent freely.***

**Name of witness_____________________**

**Signature of witness _________________**

**Date ______________________________**

**Day/month/year**

***I have accurately read or witnessed the accurate reading of the consent form to the leader or key stakeholder, and the individual has had the opportunity to ask questions. I confirm that the individual has given consent freely.***

**Name of Researcher________________________**

**Signature of Researcher ____________________**

**Date _____________________________________**

**Day/month/year**

**A copy of this Informed Consent Form has been provided to village leader _____ (initialed by the researcher/assistant)**

**Ethics Review Committee**

**Department of Medical Research**

**(Lower Myanmar)**

**Yangon, Myanmar**

**Informed Consent Form for a study of the effectiveness of repellent in preventing malaria**

This informed consent form is for men, women, and parents of children living or working in villages and worksites that are part of the National Malaria Control Program as well as Burnet Institute malaria control program in South-East Myanmar. We are inviting individuals to participate in a study looking at the effectiveness of personal insect repellent in preventing malaria. This study will also investigate markers of immune responses to malaria and malaria drug resistance. This informed consent form will be given to men, women, and parents of children who are given a rapid diagnostic test for malaria, whether they have received insect repellent or not.

**PART I: Information Sheet**

**Introduction**

We are working for the Burnet Institute and the National Malaria Control Program and we are doing research on malaria in Myanmar. We going to give you information and invite you to be part of this research. Before you decide to participate, you can talk to anyone you feel comfortable with about the research. There may be some words that you do not understand. Please ask us to stop as we go through the information and we will take time to explain. If you have questions later, you can ask them of your Malaria Officer.

**Purpose**

Malaria is a common cause of illness and death in Myanmar. Malaria is spread by mosquito bites. In Myanmar, malaria is becoming resistant to the drugs we use to treat malaria and unfortunately this resistance is spreading. One way to prevent malaria and to stop the spread of malaria drug resistance is by using insect repellent, but we want to know how effective this will be in South-East Myanmar. We would like to study whether giving personal insect repellent to mobile and migrant workers and forest dwellers helps to prevent them and others in the community from getting malaria. To do this, we would like to collect some information, perform a rapid diagnostic test for malaria, and at the same time collect a finger prick blood sample. The rapid diagnostic test will tell us if you are currently infected with malaria. The finger prick blood sample will tell us whether there are signs that you have had malaria recently. We will also look at any malaria parasite material that might be in your blood to see whether the parasite is resistant to the malaria drug artemisinin.

**Type of Research Intervention**

If you are given a rapid diagnostic test for malaria you will be invited to participate in this study. If you agree to participate, when you are given the malaria rapid diagnostic test we will collect some information about you and about the test. Immediately after the rapid diagnostic test, we will ask you to give another small sample of blood (two drops) from your finger that will be collected on a piece of filter paper. We will send this filter paper to a laboratory where we will test to see whether it contains antibodies to the malaria parasite, which may be a sign that you have had malaria recently. Immediately after the rapid diagnostic test, we will ask you to give another small sample of blood (two drops) from your finger that will be collected on a piece of filter paper. We will send this filter paper to a laboratory where we will test to see whether it contains antibodies to the malaria parasite, which may be a sign that you have had malaria recently. We will also use the blood to see whether it contains malaria parasite material (DNA). We will test this DNA to see if the parasite is likely to be resistant to the malaria drug artemisinin.

**Participant selection**

You have been chosen for this study because you are living in a village that is taking part in the National Malaria Control Program and you are going to have a malaria rapid diagnostic test. We are inviting everyone who is having a malaria rapid diagnostic test to participate in this study. If you participate in this study we will collect some information about you and the malaria test and we will take another sample of blood (two drops) from your finger for research.

**Voluntary Participation**

It is your choice whether to participate in this research study or not. Whether you choose to participate or not will not change your access to malaria testing or malaria treatment. You are free to change your mind later and stop participating in the study, even if you agreed earlier.

**Procedures and Protocol**

When you come for a malaria rapid diagnostic test, it is routine procedure for the government to collect information about your test to help show how much malaria there is in the village. We will use this routine information to study whether insect repellent provided to some people in the village/workplace helps prevent malaria in the village/workplace.

We will also ask you to give another two drops of blood onto a piece of filter paper after your finger has been pricked with a lancet for the rapid diagnostic test. If it is no longer possible to get blood from where blood was collected for the rapid diagnostic test, it may be necessary to use a lancet to prick the same finger again or a different finger to produce the two extra drops of blood for research.We will write information including the date and time, village/workplace, unique number, your age and the result of the rapid diagnostic test on the filter paper.

The blood on the filter paper will be sent to a laboratory in Australia to see whether it contains antibodies to the malaria parasite. We will also check to see whether the blood contains material from the malaria parasite and use this to test whether the parasite is likely to be resistant to the malaria drug artemisinin. The blood samples that we take from you will only be used for research purposes and will be archived after the research is completed. In future, the samples may be used for research in a related area.

**Duration**

This study will take 45 mins (or) 1 hour, but there will be no follow-up of individual participants. This means that after you have a rapid diagnostic test and provide a finger prick sample of blood on filter paper, you will not need to do anything else to be part of the study. If at a later date you are given another rapid diagnostic test you may be asked to participate in the study again.

**Risks and discomfort**

This study will involve taking small drops of blood from your finger after pricking your finger with a lancet for use in the rapid diagnostic test. This may cause minor discomfort or pain. In some cases, a second finger prick will be taken. The procedure for collecting blood for the filter paper sample is the same as for collecting blood for the malaria rapid diagnostic test. Risk of repellent includes allergy and ingestion.

**Benefits**

There may be no benefit to you personally, but your participation in this study may help us understand more about malaria in South-East Myanmar. By participating in this study you can help us work out whether insect repellent can help prevent malaria in this region and help stop the spread of drug resistance.

**Incentives**

You will not be given any money or gifts to take part in this study.

**Confidentiality**

The information that we collect from this research project will be kept confidential and no one but the researchers will be able to see it. Any information about you will have a number on it instead of your name. Only the researchers will know what your number is and we will keep it safely for a definite period.

**Sharing the Results**

The knowledge that we get from doing this research will be reported back to your village/workplace and Village Health Volunteers before it is made widely available to the public. We will publish the results and may share the results at conferences in order that other interested people may learn from our research. None of your confidential information will be shared. Individual results of rapid diagnostic test will be provided to participants after the test.

**Right to Refuse or Withdraw**

You may stop participating in the research at any time that you wish without losing your right to malaria testing and treatment. Your testing or treatment for malaria will not be affected in any way.

**Who to Contact**

If you have any questions you may ask them now or later, even after the study has started. If you wish to ask questions later, you may contact any of the following people:

Dr Win Han Oo

Phone number: 01- 375785, 375763, 375727 Ext 305 or 09 3190 6318

Burnet Institute Myanmar, No. 226, 2nd Floor, Wizaya Plaza, U Wisara Road, Bahan Township,11201 Yangon, Myanmar

This proposal has been reviewed and approved by the Ethics Review Committee, Department of Medical Research (Lower Myanmar) which is a committee whose task is to make sure that research participants are protected from harm. If you have any concern about the research, contact the secretary of the committee at the Department of Medical Research (Lower Myanmar), No 5 Ziwaka Road, Dagon PO, Yangon, phone 375457- ext: 118.

In addition, this proposal has been reviewed by the Alfred Health Human Ethics Committee, Office of Ethics & Research Governance, Ground Floor, Linay Pavilion, The Alfred,
55 Commercial Rd, Melbourne VIC 3004, Australia.

**PART II: Certificate of Consent**

I have been invited to participate in a study of the effectiveness of personal insect repellent in preventing malaria. I understand that it may or may not involve receiving insect repellent. I understand that if I choose to participate and I receive a malaria rapid diagnostic test it will involve collecting some information about me and that test. I understand that if I receive a rapid diagnostic test I will be asked to provide a small sample of blood to be collected onto a piece of filter paper. I have been informed that the risks are minimal and may include only minor discomfort associated with taking drops of blood from the finger with a lancet. I am aware that there may be no benefit to me personally and I will not be given money or gifts for participating in the study. I have been provided with the name of a researcher who can be contacted using the number and address I was given for that person.

***I have read the foregoing information, or it has been read to me. I have had the opportunity to ask questions about it and any questions that I have asked have been answered to my satisfaction. I consent voluntarily to participate as a participant in this research and understand that I have the right to withdraw from the research at any time without in any way affecting my medical care.***

**Name of Participant_______________________**

**Signature of Participant ___________________**

**Date ____________________________________**

**Day/month/year**

***If illiterate***

A literate **witness must sign** (if possible, this person **should be selected by the participant** and should have **no connection to the research team**). Participants who are illiterate should include their **thumb-print** as well.

**Thumb print of participant:**

***I have witnessed the accurate reading of the consent form to the potential participant, and the***

***individual has had the opportunity to ask questions. I confirm that the individual has given consent freely.***

**Name of witness_____________________**

**Signature of witness _________________**

**Date ______________________________**

**Day/month/year**

***I have accurately read or witnessed the accurate reading of the consent form to the potential***

***participant, and the individual has had the opportunity to ask questions. I confirm that the individual has given consent freely.***

**Name of Researcher________________________**

**Signature of Researcher ____________________**

**Date _____________________________________**

**Day/month/year**

**A copy of this Informed Consent Form has been provided to participant _____ (initialed by the researcher/assistant)**

**Ethics Review Committee**

**Department of Medical Research (Lower Myanmar)**

**Ministry of Health**

**Republic of the Union of Myanmar**

**The following information will be provided verbally to the subjects before collecting biological material from them**

**Type of sample**

If anyone comes for a malaria rapid diagnostic test, it is routine procedure for the government to collect information about the test to help show how much malaria there is in the village. We will use this routine information to study whether insect repellent provided to some people in the village/workplace helps prevent malaria in the village/workplace.

We will also ask the participant to give another two drops of blood onto a piece of filter paper after his/ her finger has been pricked with a lancet for the rapid diagnostic test. There is no additional risk for this procedure. If it is no longer possible to get blood from where blood was collected for the rapid diagnostic test, it may be necessary to make a second finger prick using a lancet. We will write information including the date and time, village/workplace, age of participants, a unique number and the result of the rapid diagnostic test on the filter paper.

The blood on the filter paper will be sent to a laboratory in Australia to see whether it contains antibodies to the malaria parasite. We will also check to see whether the blood contains material from the malaria parasite and use this to test whether the parasite is likely to be resistant to the malaria drug artemisinin. The research tests performed on the filter paper samples will not provide any information of significance to an individual’s health. The blood samples that we take from you will only be used for research purposes and any leftover sample will be archived for an indefinite period after the research is completed. Risk of using repellents includes allergy and ingestion. If you suffer side effects of repellents, you can contact your Village Health Volunteer.

**Type of consent to be obtained**

2. Unrestricted consent

If you consent some of your leftover blood samples on filter paper will be stored and may be used for further studies in a related area of research. Any new tests not covered in the present protocol will not be carried out unless a separate approval is obtained from the Ethics Review Committee, Department of Medical Research (Lower Myanmar).

**Whether identity will be retained or not?**

1. Unidentified (anonymous or anonymized).

The routine data collected will be stored in a password protected electronic database on secure servers at the Burnet Institute, Myanmar. Only the data custodians and the database manager will have access to the codes but all data and samples will be non-identifiable to data analysis staff and laboratory researchers at Burnet Institute, Melbourne, Australia. The non-identifiable data will be stored in an electronic database on the Burnet Institute network drive in Australia. Non-identifiable data is password protected on secure Institute servers and accessible to those listed on this ethics submission.

**How will confidentiality be ensured?**

The information we collect from speaking to you and from analyzing your blood samples will be kept confidential by the study team. The personal data collected at the field site will be kept confidential according to Standard Operation Procedure on data collection of Burnet Institute Myanmar. Your personal information will not be shared with anyone outside the study. No one other than the study team and authorised personnel from the study sponsor and regulatory authorities are allowed direct access to your personally identified records. When the study is completed, we will combine your test results with those of the other participants, and the overall results will be analyzed.

These overall results will be shared with other researchers at conferences and published in the medical literature. The National Malaria Control Programme will be responsible for how these data are used. In both these cases we will make sure that you cannot be personally identified. These overall results will also be shared with your village health volunteer to enable them to share the results with participants in their village. **Ethics Review Committee**

**Department of Medical Research (Lower Myanmar)**

**Ministry of Health**

**Republic of the Union of Myanmar**

**Consent for use of human blood, body fluids or tissues given for research study**

(This form should be used if the tissues/blood samples or any other human biological material will be stored for duration longer than the research study, or is likely to be used for a purpose other than mentioned in the research study.)

I consent the use of my specimen of blood for the research study entitled “Effectiveness of repellent delivered through village health volunteers on malaria incidence in artemisinin resistance containment programs”

1. I give permission for the left-over specimen to be kept for future research that is

related to this study, understanding that

my identity has been removed from the specimen ◆

**or**

my identity is kept with the specimen ◆

**OR**

2. I give permission for the left-over specimen to be kept for future research of any

type on the understanding that

my identity has been removed from the specimen ◆

**or**

my identity is kept with the specimen ◆

Name …………………………………………………………….

Signature ……………………………Date………………………….

◆ The research subject should initial or thumb print the boxes of their choice.
